# Supplementary material for: Asynchronous magnetic resonance elastography: Shear wave speed reconstruction using noise correlation of incoherent waves
Source: Magn Reson Med. 2022 Oct 27;89(3):990–1001. doi: 10.1002/mrm.29502 (PMC9792433; doi:10.1002/mrm.29502)
Supplement: Supplementary file 1 — DATA S1 MATLAB code used to generate the numerical simulations of Figure 1 [file MRM-89-990-s002.zip › k-Wave/helpfiles/k-wave_sensor_directivity.html]

Sensor Directivity (k-Wave)


# Sensor Directivity

- Example: Focussed Detector In 2D
- Example: Focussed Detector In 3D
- Example: Modelling Sensor Directivity In 2D
- Example: Modelling Sensor Directivity In 3D
- Example: Sensor Element Directivity In 2D
- Example: Focussed 2D Array With Directional Elements
